# Trends in vibrational spectroscopy on optical waveguides

**DOI:** 10.1007/s00216-026-06613-z

**Published:** 2026-06-13

**Authors:** James S. Wilkinson, Philip N. Bartlett

**Affiliations:** 1 Optoelectronics Research Centre, University of Southampton, Southampton, UK; 2 School of Chemistry and Chemical Engineering, University of Southampton, Southampton, UK

**Keywords:** Vibrational spectroscopy, Optical waveguides, Integrated photonics, Mid-infrared spectroscopy, Raman spectroscopy, Gas sensing, Biosensing, Machine learning, On-chip spectroscopy

## Abstract

Vibrational spectroscopy, due to its inherent specificity in providing a spectral “fingerprint” of molecular composition, is an important tool to identify and quantify molecules in environmental, clinical, biotechnological, security, and manufacturing process applications, for example. Optical waveguide chips, offering mass production, robustness, sensitivity and the potential for widespread, low-cost deployment as sensors, are being exploited in two principal forms of vibrational spectroscopy, waveguide mid-infrared spectroscopy (WMIRS) and waveguide-enhanced Raman spectroscopy (WERS). For both, key strands of present research include reducing waveguide attenuation, increasing light/matter interaction strength, exploring methods to deal with complex samples such as using machine learning approaches and on-chip integration of photonic devices to enhance functionality and improve performance. The mid-infrared materials and devices for WMIRS are less well developed than the near-infrared materials and devices normally used for WERS, requiring focussed research into these aspects, including new sources and detectors, and into mitigating the water absorption which can dominate parts of the MIR spectrum. In the case of WERS, additional key strands of research include reducing and mitigating the background emission from waveguide materials, enhancing the Raman signal strength, and incorporating combined plasmonic nanometallic structures with WERS. Additionally, for WERS, the issue of sample complexity hindering molecular identification is being addressed by the inclusion of Raman reporters in assays. MWIRS has been successfully applied to gas sensing, detection of compounds in breath for cancer and infection diagnosis and to bacterial discrimination in aqueous samples, for example. WERS has recently been applied to the detection of antibiotics in plasma and, for example, to cardiac biomarkers. There is now a growing trend towards commercialisation of WERS in particular, which should lead to increased application to real samples and advances in the development of practical sensors. In this review, we focus on the trends in research in WMIRS and WERS in the past 2 years.

## Introduction

Vibrational spectroscopy is a well-established technique to identify and quantify molecular components of biological, environmental and other media. This is due to its direct measurement of the frequencies and magnitudes of the vibrations of molecular bonds, which occur at frequencies between 12 and 120 THz (400 cm^−1^ < *k*_*0*_ < 4000 cm^−1^; 25 µm > *λ*_*0*_ > 2.5 µm), yielding “fingerprints” of the component molecules [1]. In principle, this allows molecular detection without the need for any additional tagging or molecular recognition element, although in complex media and with complex molecules this remains challenging.

The two principal forms of vibrational spectroscopy are (mid)infrared absorption (MIR) spectroscopy and Raman spectroscopy. These give similar but complementary information, depending on molecular symmetry, with MIR spectroscopy yielding absorptions only where bond vibrations cause changes in dipole moment and Raman spectroscopy yielding emission at frequencies shifted from incident radiation by the frequency of vibration, but only if the vibration gives rise to changes in polarisability [2]. Both show promise for compact sensors in a multitude of applications, with the principal challenges (in addition to interpretation of complex spectra) usually being strong competing background water absorption in the case of the MIR, and very weak signal against competing background noise for Raman spectroscopy.

Substantial success has been achieved in the miniaturisation of instrumentation for both techniques. In the case of MIR spectroscopy in aqueous media, for example, ATR-FTIR can be used to limit the optical pathlength through the absorbing medium, effectively to micrometre scale. This reduces water absorption, allowing measurable transmission, while also mitigating scattering from turbid media, but simultaneously lowers the absorption due to the target molecules. This trade-off means that target molecules at low concentrations often cannot be detected, and approaches such as drying samples, extraction into other solvents or surface enrichment layers are applied, allowing longer pathlengths, such as through more bulky “multi-bounce” ATR elements. In the case of Raman spectroscopy, particularly of surface layers, surface-enhanced Raman scattering (SERS) has enabled highly sensitive molecular identification and quantification, using enhanced electromagnetic fields at nanostructured metal surfaces to magnify the Raman emission by factors of typically 10^6^ on average [3]. Nonetheless, problems remain with the repeatability and robustness of SERS surfaces, rendering reliable quantification difficult.

Optical waveguides have been successfully applied to both MIR and Raman spectroscopy at surfaces. They offer the potential for further miniaturisation, mass production, small sample size, ready interfacing with optical sources and detectors on- or off-chip, long light/sample interaction lengths through the creation of spirals occupying small areas and the printing of photonic circuits integrating switches [4], filters [5] and spectrometers [6], for example. For MIR absorption spectroscopy, optical waveguides essentially represent the ultimate extension of ATR elements, providing a strong and, for monomode guides, continuous surface evanescent field. In principle, this offers very long pathlengths in small volumes of sample and thus very high sensitivity to absorption. For Raman spectroscopy, optical waveguides offer similar benefits, with long waveguide lengths offering an additional Raman enhancement without necessarily requiring the inclusion of metal nanostructures, as Raman power collected in the waveguide through the evanescent field grows linearly with length (in the absence of pump attenuation). Optical waveguide approaches do not, in themselves, offer solutions to the issues of water absorption and competition from noise and background emission, but they are well-suited to “sensorisation”, integration with microfluidics and to the inclusion of enrichment layers. Table 1 gives a comparative overview of some recent experimental demonstrations of vibrational spectroscopy on optical waveguides.

**Table 1:** Recent experimental demonstrations of vibrational spectroscopy on waveguides

**Analyte**	**WG structure**	**WG material**	**LoD**	**Focus**	**Ref**
Waveguide Mid-IR spectroscopy					
CO_2_ in N_2_	Undercut holey clad WG	SiN/Si	20 ppb (^13^CO_2_)	Isotope specific; max sensitivity	[10]
CO_2_ in N_2_	Undercut PCWG	Ge/Y_2_O_3_/Si	7.56 ppm	Long λ materials; high group index	[11]
CO_2_ in synthetic air	Integrated hollow WG	Aluminium	1.44 ppm (^13^CO_2_)	Isotope ratio to diagnose infection	[21]
CH_4_ in N_2_	Slot WG	SOI	0.3 ppm	Optimising sensitivity; minimising etalons	[16]
VOCs in breath	Integrated hollow WG	Aluminium	~ 5 ppm, VOC	Cancer detection; chemometrics	[33]
VOCs	Trenched rib	GOS	0.4 ppm for p-xylene	Enrichment layer; grating coupling	[37]
VOCs in N_2_	Suspended, SWG cladding	SOI	190 ppm, acetone/IPA	ML/AI-enhanced	[38]
VOC mixtures	Strip WG, SWG cladding	SOI	170 ppm, acetone/IPA	On-chip neuromorphic computing	[39]
VOC mixtures in water	Strip WG	SOI	2 vol%, VOC	ML/AI-enhanced; graphene detector	[27]
Bacteria in water	Thin-film WG	Diamond/AlN/Si overlayer	0.15–0.28 mg/ml (extracts)	Long λ materials; tuned for water	[34]
Mycotoxins in methanol	Thin-film WG	GaAs/AlGaAs	0.222 mg/ml (dried)	Low-power ICL for portability	[24]
Waveguide-enhanced Raman spectroscopy					
DMSO vapour in N_2_	Strip WG	Polymer sorbent, SiN/SiO_2_/Si	16.5 ppb	Device integration; fibre attached, sorbent layer	[5]
Monolayer binding studies	Slab WG and SERS	Au/Ag nanoparticles, glass WG	Submonolayer	Low-cost; plasmonic enhancement	[45]
Ceftazidime in plasma	Slot WG	Porous SiO_2_/SiN/SiO_2_/Si	< 1 mg/l	Human plasma; enhancing layer	[48]
Cardiac troponin I	Strip WG and SERRS	Au/Ag nanoparticles, AlN/SiO_2_/Si	1 nM	Sandwich assay; plasmonic enhancement	[50]

*VOC*, volatile organic compound; *ICL*, interband cascade laser; *SiN*, silicon nitride; *SOI*, silicon on insulator; *GOS*, germanium on silicon

## Waveguide mid-infrared spectroscopy (WMIRS)

**Fig. 1:**
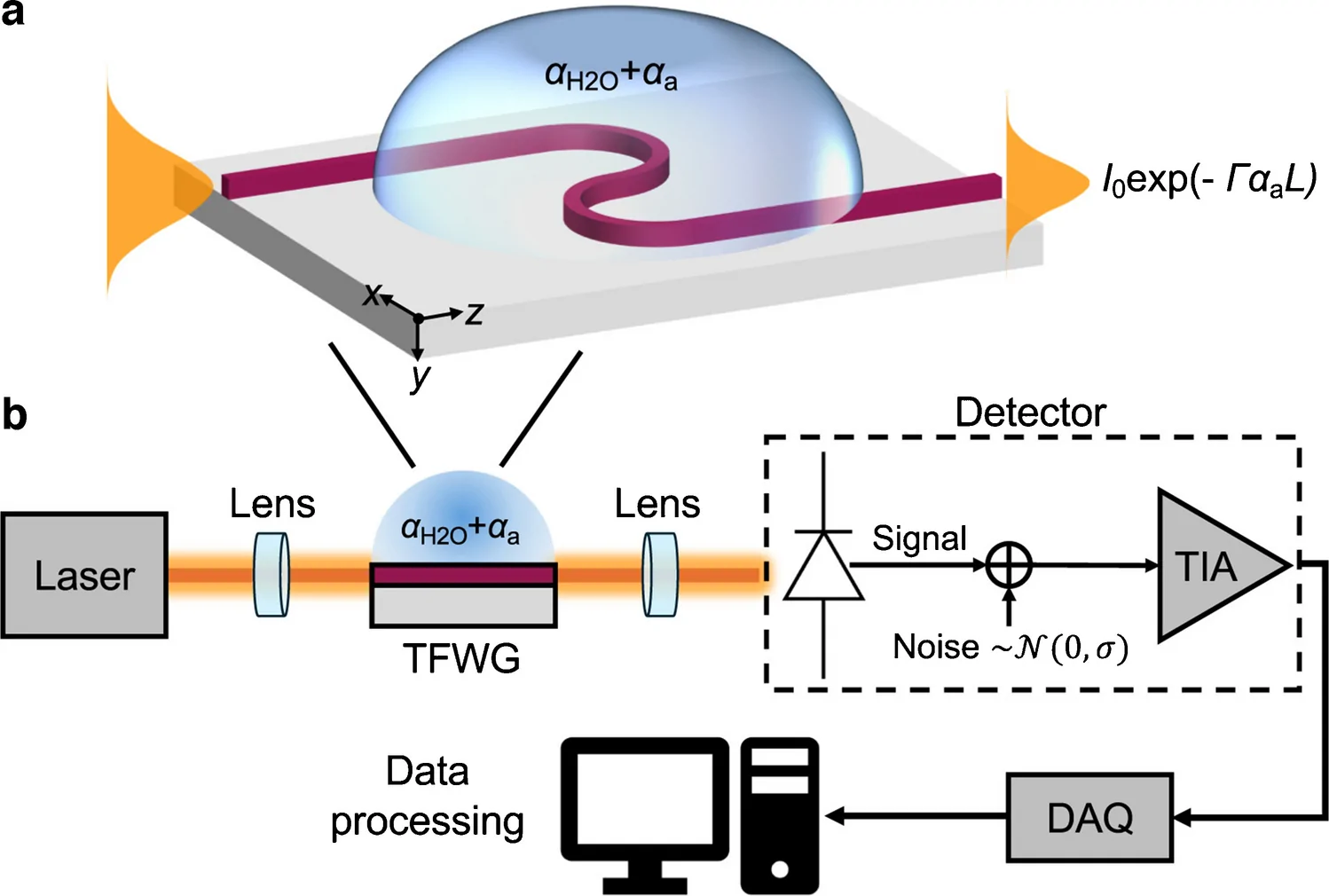
**a** Waveguide sensor for MIR absorption spectroscopy. **b** Schematic diagram of a standard spectroscopic measurement configuration. *α*_*H2O*_ and *α*_*a*_ are the absorption coefficients of water and target analyte, respectively, *I*_*0*_ is the input intensity, *Γ* is the external confinement factor and *L* is the interaction length. TFWG is the thin-film waveguide, and TIA is a transimpedance amplifier. Reprinted from [9] with permission from Elsevier

In practice, the pathlength advantage for waveguide absorption spectroscopy is severely limited by the waveguide propagation loss, partly due to the materials and processing techniques which lag those for near-infrared (NIR) applications, partly due to the trade-off involved between scattering loss and a strong surface evanescent field and partly due to water absorption, including at monolayer scale [7]. Peripheral components such as optical sources and detectors, lenses and fibres are also less well developed than their NIR counterparts. This section addresses trends since a recent comprehensive review [8] and an overview of waveguide materials and theoretical design optimisation [9]. Figure 1 shows a typical waveguide absorption spectroscopy system.

### Waveguide materials and structures

#### Undercladding materials

Silicon, silicon nitride (SiN) and germanium are the most popular waveguide core materials for MIR operation, with Si transparent at wavelengths up to about 8 µm (> 1250 cm^−1^), SiN to about 6 µm (> 2000 cm^−1^) and Ge to about 16 µm (> 625 cm^−1^). While the transparency ranges of silicon and SiN do not cover the entire MIR range of interest, silicon photonics (SiP) technology is attractive because it is very advanced and suitable for relatively short wavelength applications such as environmental gas sensing. However, waveguides need a lower-index substrate or underlayer which must also have low absorption. In conventional SiP, the undercladding is normally SiO_2_, creating “SOI” waveguides, which limits operation to <  ~ 3.5 µm, and for Ge waveguides, the substrate is often Si, creating “GOS” waveguides, which limits operation to <  ~ 8.5 µm.

Two main avenues have been pursued to address this issue: (i) suspended waveguides with substrate or undercladding locally removed and (ii) alternative undercladding materials. Salaj et al. [10] experimentally realised an undercut membrane waveguide for CO_2_ detection using a SiN core on Si, using a 2D array of holes for lateral light confinement. Operating at *λ* ≈ 4.34 µm, a limit of detection (LoD) of 20 ppb was achieved for a 2 cm long guide, despite a propagation loss of 3.4 dB/cm. This design provided very high effective “external confinement factor” of the optical power with the gas sample (*Γ*_*e*_ = 102%), very low confinement factor with the core material (*Γ*_*c*_ = 4%) and very low Fabry–Perot fringes. Note that *Γ*_*e*_ includes a “slow light” factor yielding high confinement if the group index is high, due to the waveguide structure slowing down the mode, thereby increasing sample interaction. The residual loss (*α*) was suspected to be dominated by SiN material absorption, despite the low fraction of power in the core. Kim et al. [11] experimentally realised an undercut Ge membrane waveguide on Si for CO_2_ detection with a locally etched Y_2_O_3_ undercladding transparent to ~ 13 µm, having the potential to allow integration of other devices on the remaining solid support. The waveguide losses at *λ* = 4.34 µm were high (29 dB/cm), probably due to fabrication imperfections, but the effective pathlength was slightly enhanced due to the slow-light effect of the lateral cladding hole array.

Suspended waveguides are sometimes considered fragile, particularly for application to liquid samples, so solid undercladding materials with long wavelength transmission are being pursued. A promising approach is the growth of SiGe with a graded composition between the high-Ge core layer and the underlying Si, which ensures that very limited optical power interacts with the Si, leading to losses below 1 dB/cm for 5 µm < *λ* < 8.5 µm in spiral waveguides up to 14 cm long [12]. These were demonstrated on 200 mm Si wafers in a step towards mass production, but evanescent interaction with an analyte was not an object of the study.

Additional approaches to enhance absorption, allowing shorter waveguides and mitigating propagation loss, include surface-enhanced infrared absorption (SEIRA) and polymer surface enrichment layers [13]. SEIRA employs a nanostructured metal surface, similar to that used in SERS, and recent reports on SEIRA surfaces show improvements in both theoretical understanding and experimental performance [14, 15], which may improve applicability to WMIRS.

#### Slot waveguides

Slot waveguides, formed like rib waveguides but with a ~ 100–200 nm wide slot running down their centres, have seen significant application to MIR absorption spectroscopy because they concentrate the modal field in the sample-filled gap (gas or liquid) and thereby yield a large *Γ*_*e*_. While in the NIR slot waveguides suffer in comparison with simple strip waveguides due to scattering loss at MIR wavelengths, these should be less dominant. Yallew et al. [16] experimentally realised a silicon-on-insulator (SOI) slot waveguide operating around *λ* ≈ 3.27 nm, with *Γ*_*e*_ = 67% and achieving an LoD for methane of < 300 ppb. Propagation losses were of order 10 dB/cm, higher than the 1–6 dB/cm expected, which was believed to be due to water absorption from the environmental gas sample. Note that low LoD in these and the above reports results from careful management of noise and Fabry–Perot fringe reduction and good data processing, as well as optimised device design, and that the SOI structure would be unlikely to allow CO_2_ measurements around 4.3 µm. Slot waveguides show lower confinement factors than the suspended membrane waveguides above, and ultimately, a trade-off must be reached between confinement factor, loss and Fabry–Perot interferences, and technologically the devices must be mass-manufacturable and the materials ideally compatible with silicon processing.

#### Waveguide in-coupling and out-coupling

Practical application of MIR waveguide sensors requires in- and out-coupling with low loss and good alignment tolerance. The ultra-low core confinement exhibited by the suspended waveguide design demonstrated in [10] results in high end-fire coupling efficiency, due to the size and shape of the mode. Grating in/out couplers are often used in silicon photonics because they allow positioning anywhere on the chip, and no special care with end-facets, but have the potential disadvantage of narrower-band operation. A compelling experimental demonstration of this uses microlenses on the underside of the silicon substrate, coupling light through the substrate into and out of the GOS waveguide using gold-coated grating couplers [17].

#### Optofluidic sensing and hollow waveguides

Optofluidic techniques to process samples and deliver them to waveguides for spectroscopy are beyond the scope of this article and are described in detail in [18]. However, integrated hollow waveguides (iHWG), where light is guided through a channel filled with a sample, with maximum field strength located in the sample, rather like slot waveguides but with a different light-guiding mechanism, relaxed fabrication and normally stronger light-sample interaction, are showing promise [19]. Recently, an aluminium iHWG with IR-transparent end-caps and inlet/outlet for gas samples was used to measure CO_2_ isotopes in breath for potential gastrointestinal disease diagnosis [20], achieving a LoD as low as 1.44 ppm. The internal surfaces of the aluminium “light-pipes” of mm-scale radius and ml-scale sample volume were polished to < *λ*/10 quality, allowing low-loss guidance by (high but non-total) internal reflection from the metallic walls. This type of waveguide is very highly multimoded, which allows ready use of incoherent sources and mitigates against significant modal noise, and the large diameter ensures low loss through low interaction of light with the sidewalls. While this approach may not be as suitable for mass production as the integrated photonic waveguides described above, it uses low-cost fabrication techniques and benefits from simplicity and practicality, rendering iHWGs an intriguing trend.

### Optical sources and detectors for WMIRS

Thermal sources such as glo-bars are usually used for conventional MIR ATR spectroscopy, being stable, low-cost and broadband. However, the power which can be end-coupled into a single waveguide mode from a thermal source is fundamentally limited. Higher-brightness alternatives such as quantum cascade lasers (QCLs), interband cascade lasers (ICLs), LEDs and supercontinuum sources are being pursued, as reviewed in detail in [8]. Off-chip QCLs are most often used in research, but are expensive and power-hungry, although there have been recent experimental demonstrations of monolithic and hybrid integration of QCLs with MIR waveguides for robust, integrated on-chip spectroscopy [21, 22]. ICLs exhibit low power consumption in comparison with QCLs and, despite worse noise performance than a conventional ATR-FTIR system, a tunable ICL/waveguide system with mercury cadmium telluride (MCT) detector exhibited improved LoD for the food contaminant deoxynivalenol, primarily because of the much longer effective pathlength enabled by higher coupled power [23]. The wavelength tuning range of QCLs and ICLs is limited in comparison with the bandwidth of thermal sources, and their applicability will depend on the spectral range required, though note that a broadband source requires a spectrometer. A novel approach to realising a supercontinuum source, which produces high-brightness broadband emission from very short pump pulses through nonlinear interactions, was demonstrated in [24] where waveguides with SiGe graded claddings similar to [12] produced a spectrum stretching from 5 to 13 µm from input laser pulses (*P*_*av*_ = 1.6 mW, *λ* = 9 µm). This exploited high nonlinearity, strong core confinement and well-designed dispersion properties and paves the way for pulsed QCL-driven operation. Thermal infrared emitters, using thin electrode films such as graphene, have been integrated on silicon-based waveguides, enabling near-field coupling of broadband thermal emission into the waveguides through evanescent fields [25]. This near-field approach allows coupling of greater power into an individual mode than the end-fire configuration and shows promise for low-cost integration of compact on-chip sources where broadband operation is required and there is low background absorption and low propagation loss. Overall, the choice of source for waveguide MIR absorption spectroscopy is highly application-dependent, with ICLs seeming poised to be frontrunners.

In general, external MCT detectors are used in WMIRS, but the on-chip integration of detectors for improved robustness, simplicity and flexibility of detector placement is a growing trend. Examples of this are (i) graphene waveguide-integrated photodetectors and (ii) thin-film waveguide-integrated bolometers. The former integrates the graphene film on a SOI waveguide by exfoliation and dry transfer, followed by electrode patterning, and achieved an NEP of 7.5 nW/Hz^½^ experimentally [26] while the latter utilised conventional CMOS processing techniques to monolithically integrate a Ti/TiO_x_ bolomet on a GOI waveguide, achieving an experimental NEP of 27 nW/Hz^½^ [27]. While these noise figures are not as low as those of discrete commercial MCT devices, detector noise is often not the limiting factor in determining LoD.

### Applications of waveguide mid-infrared spectroscopy

Applications of WMIRS have been covered in three recent reviews, focussing on biomedical applications [8, 28] and environmental and volatile organic compounds (VOCs) [8, 29]. Applications published over the past 2 years fall into four broad areas: gas detection and monitoring, which is the most popular, followed by biological/biomedical, then detection of volatile organic compounds (VOCs) and finally applications combining MIR waveguides with machine learning.

In the area of gas detection and monitoring, the most common applications have been on the use of different MIR waveguide architectures, experimentally demonstrating sub-ppm LoD [16] and theoretically studying improvements in sensitivity [30] or LoD [31]. Most of the work has focussed on CO_2_ measurement, but there is also work on other gases, typically CH_4_, CO and N_2_O, and on discriminating between them.

**Fig. 2:**
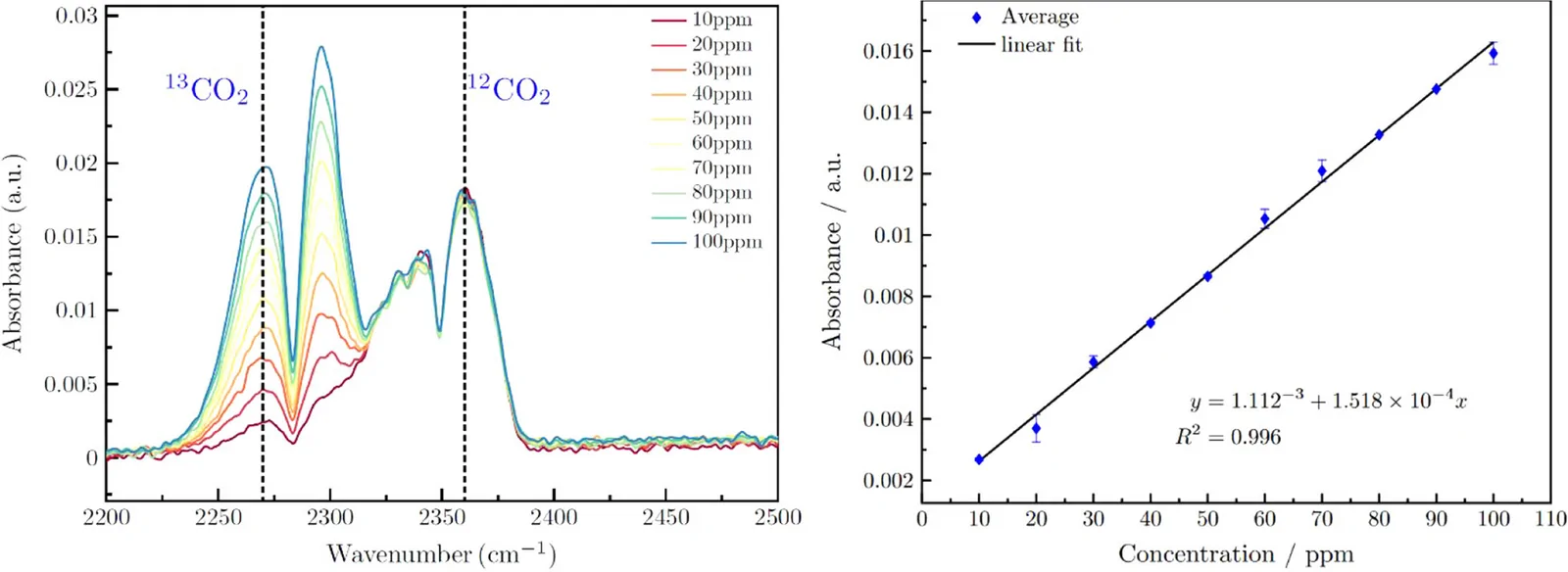
Left: IR spectra of ^13^CO_2_/^12^CO_2_ ratios in 10 cm iHWGs with ^12^CO_2_ fixed at 200 ppm and ^13^CO_2_ concentrations varying from 10 to 100 ppm. Right: Calibration curve as a function of ^13^CO_2_ concentration. From Ref [20] under CC BY 4.0

In contrast to the detection of simple molecules in the gas phase, for biomedicine the challenge lies in the inherent complexity and sample-to-sample variability. This is particularly true of medical diagnostic applications using breath sampling. Glocker et al. [32] combined an array of two types of gas sensors (an eNose module) with a MIR waveguide to discriminate between gastric cancer patients and healthy individuals, based on the pattern of exhaled VOCs. To reduce extraneous background variations, the study participants were subject to very stringent behavioural constraints for 2 h before the measurement. An alternative approach to medical diagnostic applications is the use of a specific marker compound, for example acetone in the breath [19]. This approach has been used to detect *Heliobacter pylori* infection using ^13^CO_2_ [20]. The method is based on the detection of ^13^CO_2_ following the administration of a gel or pill containing ^13^C-labelled urea. The urea is metabolised by the *H. pylori* bacteria in the upper gastrointestinal tract, releasing ^13^CO_2_ which is detected on the patient’s breath. The method requires measurement of the ^13^CO_2_-to-^12^CO_2_ ratio (as shown in Fig. 2) down to around 0.6% which is feasible using MIR waveguides [10, 20]. The identification of different types and strains of bacteria poses similar challenges in terms of complexity and variability. In a proof of concept study, Angelstan et al. [33] have shown that MIR waveguides can be used to discriminate between *A. aureus*, a gram-positive bacterium, and *E. Coli*, a gram-negative bacterium, based on the characteristic MIR spectrum of the lipopolysaccharides that are found in the bacterial cell wall. In biological applications, detection of a specific marker compound amidst a complex mixture can also be useful, for example as a marker for fungal infection [23].

Polymer surface enrichment layers concentrate target molecules in the evanescent field and can exclude water, increasing sensitivity, reducing sample background absorption and rendering the platform more application-specific. Methane sensing using cryptophane-doped polymer membranes has been demonstrated with refractometric sensors but so far suffers from poor specificity; combination with spectral fingerprinting may solve this problem in WMIRS [34]. Detection and measurement of VOCs, typically aromatic hydrocarbons such as benzene, toluene and xylene, present as pollutants in water is a significant application for MIR waveguides where the use of hydrophobic polymer cladding is beneficial to concentrate the VOC on the waveguide surface and reduce the interference from water [13, 35]. In the gas phase, an ordered mesoporous silica film has been used for the same purpose [36].

A growing trend is combining WMIRS with machine learning. In this context, the work of Lee’s group is interesting. Building on their earlier work on the application of AI to MIR waveguide analysis of liquid mixtures, in the past 2 years, they have incorporated a zero-bias graphene photodetector with the waveguide and demonstrated the application of this to the analysis of mixtures of 1–5% IPA, acetone and ethanol in water using a convolutional neural network to classify the mixtures and a convolutional regression network to decompose the spectra [26]. A similar approach was used to demonstrate discrimination of binary mixtures of IPA and acetone in pure nitrogen [37] and taken one stage further through the incorporation of neuromorphic in-sensor computing [38]. In this case, the photoresponsivity of the on-chip graphene photodetector was used in the weighting for the artificial neural network, and this photoresponsivity was tuned by adjusting the bias voltage.

## Waveguide-enhanced Raman spectroscopy (WERS)

**Fig. 3:**
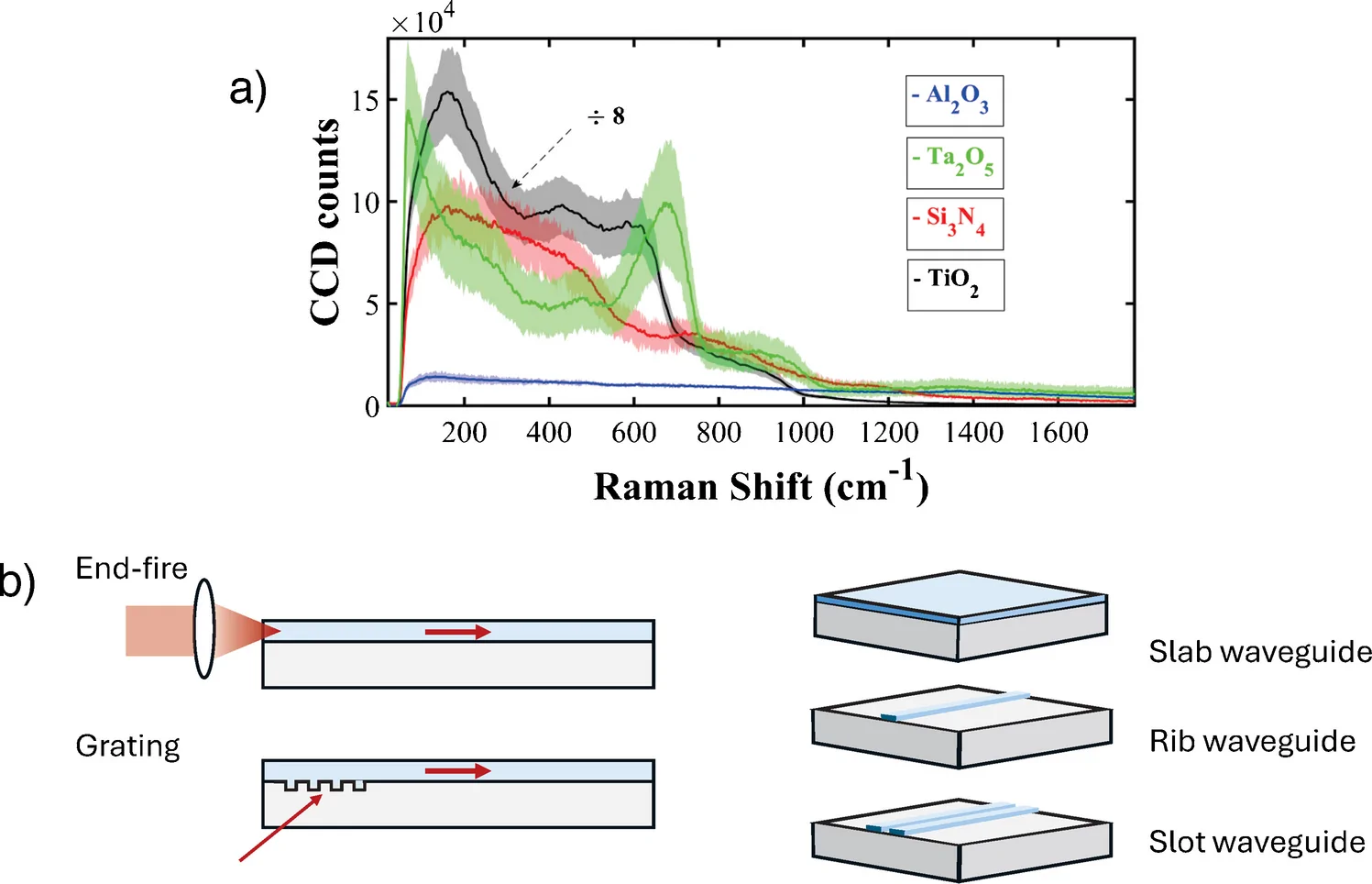
**a** Waveguide background emission; reprinted with permission from [41] © Optica Publishing Group. **b** Laser/chip in-coupling methods and basic structures

Waveguide-enhanced Raman spectroscopy benefits from the ability to build up a strong Raman signal with waveguide length, without requiring fragile nanostructured metals, and from highly developed optoelectronic materials and components in the NIR. However, due to the short wavelengths used and the need for high surface fields, the useful waveguide length is severely limited by waveguide propagation loss. LoDs are also fundamentally limited by noise, as always, and in particular by shot noise on background emission from the waveguides (Fig. 3a) and sample media. Thus, device research priorities include materials, processes and device structures for low loss and background reduction or signal enhancement. This section addresses advances since recent reviews of WERS [39, 40].

### Waveguide materials and structures

The predominant waveguide core material for evanescent WERS is silicon nitride (SiN) because of its good (but not unique) performance and, perhaps more significantly, because of its technological maturity [41]. The pump wavelength of choice is normally 785 nm, representing a compromise between background fluorescence and strength of Raman signal, although shorter wavelengths are required if large Raman shifts are to be measured using silicon detectors [42]. Collecting Raman emission in the backscattering configuration (wrt pump propagation) [42] is preferred due to a reduced need for pump rejection and a relaxed tolerance on waveguide length. While slot waveguides have the advantage of lower waveguide background emission, because less power travels in the waveguide core, increased propagation loss compared with simple rib waveguides often outweighs this advantage. Because very compelling basic designs (wavelength, materials, structures) have emerged, the primary focus is now on refining performance. For example, detailed work has been carried out on separating core and cladding contributions to waveguide background emission and minimising propagation loss in SiO_2_-clad SiN waveguides, with the aim of establishing optimum processes and designs [43] for foundry production. There also remains a distinct strand of research aiming to combine the benefits of plasmonics (as in SERS) with those of dielectric waveguides [44], for further signal enhancement or reduced background emission.

In contrast to molecular sensing in solutions, analysis of individual micro- and nano-sized biological particles does not require evanescent spectroscopy, and lower-index-contrast waveguides are proposed to deliver and collect power to and from stationary particles using field projection [45]. This study confirmed sources of background emission in dielectric waveguides and showed low background emission from UV-written silica waveguides.

### Device integration

The potential of integrated photonics for increased on-chip functionality is being exploited to integrate devices on chip without, in general, increasing fabrication complexity. Lattice filters to separate pump and signal and edge couplers for low-loss fibre attachment were integrated with waveguide spirals and a polymer sorbent layer to demonstrate robust integration of multiple components on chip [5], in one of the most complete WERS system demonstrations. Integrated spectrometers in SiN waveguide chips are being very actively pursued, though few have been integrated with WERS. Most demonstrations of WERS employ external spectrometers which, while becoming more compact, are still bulky compared to the WERS chip and pump laser. A neat hybrid approach, which integrates the dispersive components on chip with a 32-waveguide optical phased array and 32 grating out-couplers, while Fourier imaging in free space onto a cooled camera to obtain the 2D angular (and hence spectral) distribution, has been demonstrated [6]. The spectrometer operated over Raman shifts up to 1200 cm^−1^ with a resolution of about 8 cm^−1^, suitable for structurally simple analytes in liquid samples, with a 0.12 mm^2^ on-chip footprint and with design potential for improved resolution and spectral coverage. Integration of high-Q waveguide resonators is of potential interest for signal enhancement, potentially minimizing sample volume, and micro-ring resonators have been used to further enhance WER spectra from graphene layers [46], and future application to organic materials is mentioned.

### Applications of waveguide-enhanced Raman spectroscopy

As with WMIRS, the use of adsorption layers to concentrate analytes at the waveguide surface, increasing sensitivity and selectivity, continues to attract attention both for vapour-phase detection [5] and solution applications. Thus, Zhao et al. [47] demonstrated the detection of the antibiotic ceftazidime (molecular weight 547 Da) in plasma in the mg/L range using WERS on a SiN waveguide modified with a mesoporous silica film. The 4 nm pores in the silica layer exclude large interferent molecules in the plasma.

For surface-enhanced Raman spectroscopy, first demonstrated in the 1970 s, the main contribution to the very significant (4 to 6 orders of magnitude) surface enhancement observed for nanostructured coinage metals (Cu, Ag, Au) is the increase in the electromagnetic field at the metal surface by the excitation of surface plasmon resonances [3]. The idea of combining plasmonic nanometallic structures with waveguides and using the waveguide to excite the surface plasmons is thus an intriguing and exciting area for study. Gao et al. [48] have recently reviewed the different types of SERS-integrated optical waveguides and their application to the measurement of trace species in solution for applications ranging from medical diagnostics to environmental monitoring. In their review, they highlight the significant potential benefits of combining the surface-enhanced Raman spectroscopy with waveguide optics. Two recent publications illustrate this trend:

Chen et al. [44] use a thick (200 μm) slab waveguide made from a microscope cover slip. This supports around 800 TE and 800 TM modes so that it acts like an attenuated total reflectance (ATR) guided-wave device. It has the advantage of being cheap, readily available and thus disposable. This waveguide is modified by forming a nanoparticle matrix of ~50 nm Au@Ag core-shell particles bound to the surface by (3-aminopropyl) triethoxysilane (APTES) modification of the glass surface. The slab waveguide is used to excite SERS for molecules adsorbed on the surface of the Au@Ag nanoparticles. As a demonstration, they show spectra for 4-mercaptopyridine (4MPy) and 4-aminobenzoic acid, recorded for TE and TM modes.

**Fig. 4:**
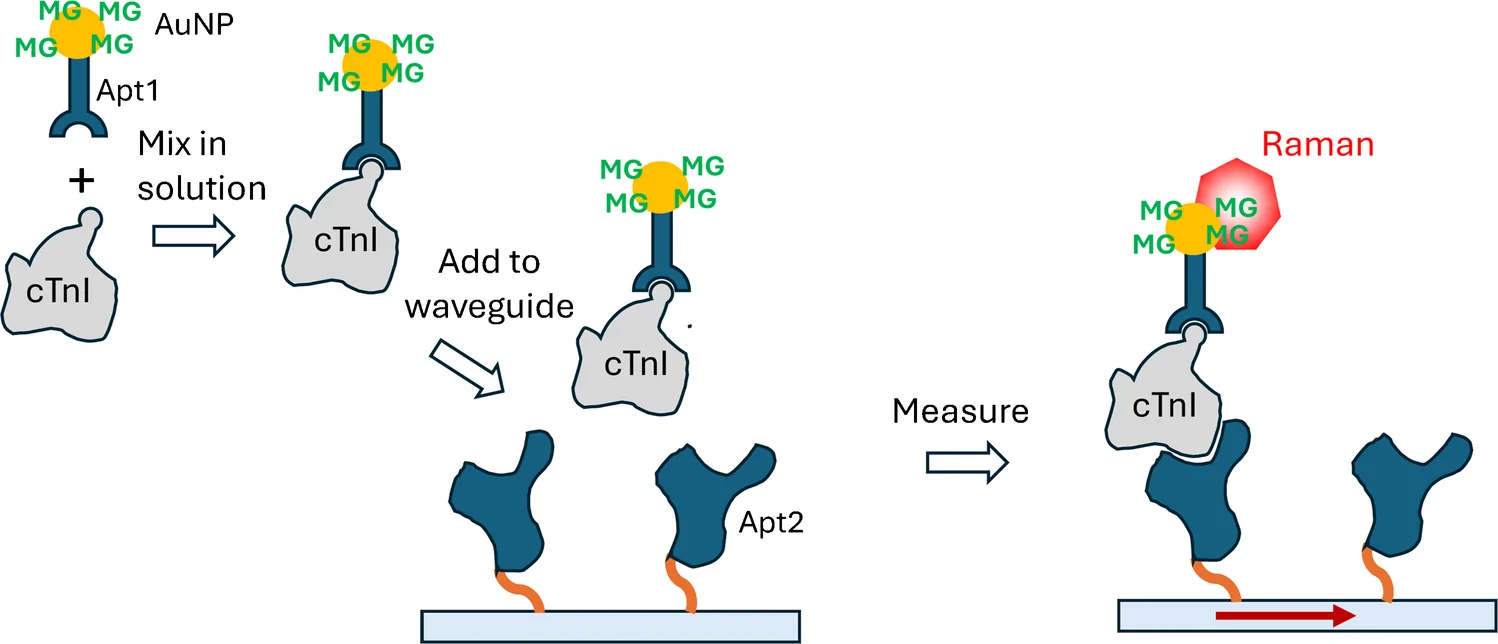
Aptamer sandwich assay format for cTnI using a combined WERS/SERS device [49]

Makela et al. [49] have combined the use of SERS with WERS to demonstrate the detection of the 24 kDa protein cardiac troponin I (cTnI), a blood cardiac biomarker for the diagnosis of myocardial infarction. They used an aptamer sandwich assay on the waveguide surface, as shown in Fig. 4, using a gold nanoparticle label to provide the required selectivity and sensitivity (the clinically relevant range for cTnI is 0.1 to 1.5 ng/ml). The first aptamer is bound to the waveguide surface through bonding to (3-aminopropyl) trimethoxysilane (APTMS) and glutaraldehyde. In the assay, the cTnI binds to a second aptamer that has been labelled with 36 nm gold nanoparticles which have malachite green isothiocyanate (MGITC) bound to their surface. Then, when brought into contact with the modified waveguide surface, the cTnI also binds to the surface-bound aptamer (hence sandwich assay), and the whole assembly is detected by measurement of the enhanced Raman spectrum of the MGITC on the AuNPs excited by evanescent 635 nm light from the waveguide. The Raman reporter MGITC absorbs at 620 nm, so there is also a resonant contribution to the enhancement (WERS excited surface-enhanced *resonance* Raman spectroscopy or SERRS). Since the dye has four sharp, characteristic Raman bands, it is easily discriminated in the spectrum.

### Outlook

Direct comparison of the performance of WMIRS and WERS is hampered by the different tasks to which they have been applied and the imbalance in the development of materials and devices in the MIR compared with the NIR/VIS, as reflected in the level of research activity on these topics.

WMIR spectroscopy is well-suited to environmental monitoring of gases, especially if waveguide propagation losses and those due to water adsorption can be further reduced. These applications have the advantage of not requiring chip disposal between each measurement, allowing for more costly chips and more permanent interfacing. WMIR measurements in biochemical applications suffer from the complexity of the molecules targeted and the (usually aqueous) matrix, which is likely to require drying and/or significant sample pre-treatment. Nonetheless, the success of conventional ATR-FTIR in combination with ML, for example in cancer diagnostics [50], bodes well for WMIRS if chip interchangeability (for disposability) is simplified, if sample pre-treatment is minimised and integrated and, ideally, with enhanced ML explainability.

Raman spectroscopy has the benefit of emission frequencies separated from the excitation frequency, providing a noise advantage, and SERS can also offer well-developed biochemical assay protocols to WERS. While an aqueous matrix poses little problem for WERS, matrix and molecular complexity is still an issue, but strong progress on SERS has shown that appropriate labelling with Raman reporters, for example, can yield high performance and mitigate surface variability, approaches which could be transferred to WERS (with or without plasmonics) at the cost of the elusive label-free operation. Alternatively, AI/ML-assisted approaches may achieve good performance without labelling [51].

For both WMIRS and WERS, most demonstrations have used much-simplified model chemical systems. This was important to establish the photonic technologies, but now that the devices are reasonably advanced, collaborative research with application specialists and users, particularly in biomedical fields, should be accelerated to establish real applicability and determine fields where the greater complexity of using these devices is surmounted by their promise of improved functionality, performance, robustness, compactness or cost. The recent establishment of start-up companies in WERS, in particular, should meet some of this need and emphasises the more advanced stage of this technology.

## References

[1] Baker MJ, Hussain SR, Lovergne L, Untereiner V, Hughes C, Lukaszewski RA, et al. Developing and understanding biofluid vibrational spectroscopy: a critical review. Chem Soc Rev. 2016;45(7):1803–18. 10.1039/c5cs00585j 26612430

[2] Banwell CN. Fundamentals of molecular spectroscopy. 3rd ed: McGraw-Hill, London 1983.

[3] Yi J, You EM, Hu R, Wu DY, Liu GK, Yang ZL, et al. Surface-enhanced Raman spectroscopy: a half-century historical perspective. Chem Soc Rev. 2025;54(3):1453–551. 10.1039/d4cs00883a 39715320

[4] Qi Y, Zheng Z, Banakar M, Wu Y, Gangnaik A, Rowe D, et al. Integrated switching circuit for low-noise self-referenced mid-infrared absorption sensing using silicon waveguides. IEEE Photon J. 2021;13(6):1–10. 10.1109/jphot.2021.3121331

[5] Tyndall NF, Emmons ED, Pruessner MW, Butt JN, Walsh KJ, Hung KC, et al. Waveguide-enhanced Raman spectroscopy of trace vapors with fiber-attached photonic integrated circuits. Chem Biol Radiol Nucl Explos (CBRNE) Sensing XXV2024. 10.1117/12.3012880

[6] Liu Z, Le Thomas N, Baets R. Broadband, high resolution, sensitive spectrometer using an integrated optical phased array in silicon nitride and fourier imaging. J Light Technol. 2025;43(3):1306–11. 10.1109/jlt.2024.3483556

[7] Jágerská J, Torres-Cubillo A, Feiler M, Zakoldaev R, Salaj J, Mestre N, et al. Water monolayers: an overlooked loss mechanism in mid-infrared photonics (Preprint). Res Square. 2026. 10.21203/rs.3.rs-9284522/v1

[8] Petruci JFD, Sousa DD, Mizaikoff B. Advanced mid-infrared sensors for molecular analysis. Anal Chem. 2025;97(13):6871–90. 10.1021/acs.analchem.4c06799 PMC11983364 40151985

[9] Torres-Cubillo A, Teuber A, Halir R, Mizaikoff B. Optimized waveguides for mid-infrared lab-on-chip systems: a rigorous design approach. Sensors Actuators A Phys. 2024;378. 10.1016/j.sna.2024.115797

[10] Salaj J, Vlk M, Zakoldaev R, Seton R, Čtyroký J, Alberti S, et al. Suspended nanophotonic waveguide for isotope-specific CO_2_ detection. Optica. 2024;11(12). 10.1364/optica.533710

[11] Kim I, Lim J, Shim J, Park J, Ahn SY, Lim H, et al. Freestanding germanium photonic crystal waveguide for a highly sensitive and compact mid-infrared on-chip gas sensor. ACS Sens. 2024;9(10):5116–26. 10.1021/acssensors.4c00941. 39410757

[12] Turpaud V, Nguyen TH, Dely H, Koompai N, Bricout A, Hartmann JM, et al. Low-loss SiGe waveguides for mid-infrared photonics fabricated on 200 mm wafers. Opt Express. 2024;32(10):17400–8. 10.1364/OE.521925 38858924

[13] Baillieul M, Rinnert E, Lemaitre J, Michel K, Colas F, Bodiou L, et al. Surface functionalization with polymer membrane or SEIRA interface to improve the sensitivity of chalcogenide-based infrared sensors dedicated to the detection of organic molecules. ACS Omega. 2022;7(51):47840–50. 10.1021/acsomega.2c05502. PMC9798758 36591173

[14] Colleu T, Fekete A, Gonze X, Cloots A, Liégeois V, Rignanese GM, et al. Surface enhanced infrared absorption mechanism and modification of the plasmonic response. J Phys Photonics. 2024. 10.1088/2515-7647/ad2529.

[15] Li DX, Zhou H, Ren ZH, Xu C, Lee C. Tailoring light-matter interactions in overcoupled resonator for biomolecule recognition and detection. Nano-Micro Lett. 2025;17(1). 10.1007/s40820-024-01520-3 PMC11427657 39325238

[16] Yallew HD, Vlk M, Datta A, Alberti S, Zakoldaev RA, Hovik J, et al. Sub-ppm methane detection with mid-infrared slot waveguides. ACS Photon. 2023;10(12):4282–9. 10.1021/acsphotonics.3c01085 PMC10740002 38145165

[17] Frank F, Verstuyft M, Teigell Beneitez N, Missinne J, Roelkens G, van Thourhout D, et al. Experimental demonstration of the high alignment-tolerant behavior of a mid-infrared waveguide platform for evanescent field sensing. ACS Appl Opt Mater. 2024;2(9):1926–32. 10.1021/acsaom.4c00280. PMC11443523 39360237

[18] Testa G, Persichetti G, Bernini R. Optofluidic biosensing: devices, strategies, and applications. TrAC Trends Anal Chem. 2024. 10.1016/j.trac.2024.117865.

[19] Barreto DN, Schreiber C, da Silveira Petruci JF, Mizaikoff B, Kokoric V. Mid-infrared acetone gas sensors using substrate-integrated hollow waveguides augmented by advanced preconcentrators. Sci Rep. 2025;15(1):18855. 10.1038/s41598-025-02514-w. PMC12122893 40442212

[20] Rangel GF, Martinez LDD, Mizaikoff B. *Helicobacter pylori* breath test via mid-infrared sensor technology. ACS Sens. 2025;10(2):1005–10. 10.1021/acssensors.4c02785 PMC11877511 39921651

[21] Wang R, Täschler P, Wang Z, Gini E, Beck M, Faist J. Monolithic integration of mid-infrared quantum cascade lasers and frequency combs with passive waveguides. ACS Photon. 2022;9(2):426–31. 10.1021/acsphotonics.1c01767

[22] Wang DB, Kannojia HK, Jouy P, Giraud E, Suter K, Maulini R, et al. Innovative integration of dual quantum cascade lasers on silicon photonics platform. Micromachines-Basel. 2024;15(8). 10.3390/mi15081055 PMC11356703 39203706

[23] Femenias A, Erdem M, Fomina P, Scheuermann J, Blazhko U, Freitag S, et al. Portable tunable interband cascade laser spectrometer using thin-film waveguides for food contaminant analysis. AIP Adv. 2024;14(12). 10.1063/5.0226248

[24] Turpaud V, Yang Y, Dely H, Bricout A, Nguyen THN, El Bouchikhi N, et al. Low-power supercontinuum generation in Ge-rich SiGe waveguides from 4 to 13 μm wavelength. APL Photonics. 2025;10(9). 10.1063/5.0270228

[25] Negm N, Zayouna S, Parhizkar S, Lin PS, Huang PH, Suckow S, et al. Graphene thermal infrared emitters integrated into silicon photonic waveguides. ACS Photonics. 2024;11(8):2961–9. 10.1021/acsphotonics.3c01892 PMC11342416 39184180

[26] Zhou JK, Liu XM, Zhou H, Xu SY, Xie JS, Xu C, et al. Artificial-intelligence-enhanced mid-infrared lab-on-a-chip for mixture spectroscopy analysis. Laser Photonics Rev. 2025;19(1). 10.1002/lpor.202400754

[27] Kim I, Shim J, Lim J, Jeong J, Kim BH, Kim S. Highly sensitive free-standing waveguide-integrated bolometer on germanium-on-insulator platform for mid-infrared on-chip spectroscopy. IEEE Transact Electron Devices. 2025;72(5):2733–9. 10.1109/ted.2025.3535465

[28] Rangel GF, Martínez LDD, Walter LS, Mizaikoff B. Recent advances and trends in mid-infrared chem/bio sensors. TrAC-Trends Anal Chem. 2024;180. 10.1016/j.trac.2024.117916

[29] Xia L, Liu Y, Chen RT, Weng B, Zou Y. Advancements in miniaturized infrared spectroscopic-based volatile organic compound sensors: a systematic review. Appl Phys Rev. 2024;11(3). 10.1063/5.0197236

[30] Butt MA, Piramidowicz R. Suspended slot membrane waveguide based on germanium-on-silicon-on-insulator at lambda = 4.23 microns for CO_2_ monitoring. Micromachines (Basel). 2024;15(12). 10.3390/mi15121434 PMC11678585 39770188

[31] Benkohaila N, Lorrain N, Bahsine S, Lmai F, Charrier J. Design of an optical gas sensor based on chalcogenide (ChG) glass platform in the mid-infrared for detection of CO_2_ and CO. Opt Quant Electron. 2024;56(10). 10.1007/s11082-024-07486-1

[32] Glockler J, Mitrovics J, Beeken S, Leja M, Welearegay T, Osterlund L, et al. Infrared spectroscopic electronic noses: an innovative approach for exhaled breath sensing. ACS Sens. 2025;10(1):427–38. 10.1021/acssensors.4c02725. PMC11773567 39772459

[33] Angelsten A, Forsberg P, Engqvist H, Xia W, Karlsson M. Waveguide spectroscopy for differentiation of bacteria. Anal Chem. 2025;97(39):21314–23. 10.1021/acs.analchem.5c02661. PMC12509186 40999861

[34] Alberti S, Brotin T, Jagerska J. Rethinking cryptophane-A for methane gas sensing: cross-sensitivity to N2 and CO2 at ambient conditions. Anal Chem. 2025;97(40):21793–8. 10.1021/acs.analchem.5c03869 PMC12529469 41026513

[35] Vrazel M, Ismail RK, Courson R, Hammouti A, Bouska M, Larrode A, et al. Surface functionalization of a chalcogenide IR photonic sensor by means of a polymer membrane for water pollution remediation. Analyst. 2024;149(18):4723–35. 10.1039/d4an00721b 39105485

[36] Frank F, Baumgartner B, Verstuyft M, Teigell Beneitez N, Missinne J, Van Thourhout D, et al. Integrated optics waveguides and mesoporous oxides for the monitoring of volatile organic compound traces in the mid-infrared. Appl Spectrosc. 2025;79(5):842–51. 10.1177/00037028241300554 PMC12053257 39692077

[37] Liu XM, Zhang ZX, Zhou JK, Liu WX, Zhou GY, Lee CK. Artificial intelligence-enhanced waveguide “photonic nose”- augmented sensing platform for VOC gases in mid-infrared. Small. 2024;20(34). 10.1002/smll.202400035 38576121

[38] Liu X, Zhang Z, Zhou J, Liu W, Zhou G, Lee C. Development of photonic in-sensor computing based on a mid-infrared silicon waveguide platform. ACS Nano. 2024;18(34):22938–48. 10.1021/acsnano.4c04052 39133149

[39] Ettabib MA, Marti A, Liu Z, Bowden BM, Zervas MN, Bartlett PN, et al. Waveguide enhanced Raman spectroscopy for biosensing: a review. ACS Sens. 2021;6(6):2025–45. 10.1021/acssensors.1c00366. 34114813

[40] Ettabib MA, Liu Z, Zervas MN, Bartlett PN, Wilkinson JS. Waveguide-enhanced Raman spectroscopy. Nat Rev Methods Prim. 2024;4(1). 10.1038/s43586-023-00281-4

[41] Raza A, Clemmen S, Wuytens P, de Goede M, Tong ASK, Le Thomas N, et al. High index contrast photonic platforms for on-chip Raman spectroscopy. Opt Express. 2019;27(16):23067–79. 10.1364/Oe.27.023067 31510589

[42] Tyndall NF, Emmons ED, Pruessner MW, Rabinovich WS, Wilcox PG, Tripathi A, et al. Foundry-based waveguide-enhanced Raman spectroscopy in the visible. Opt Express. 2024;32(4):4745–55. 10.1364/oe.504195. 38439219

[43] Garcia Coleto A, Crawford-Eng H, Tyndall N, Antohe A, Kar S, Carpenter L, et al. Extracting the core and cladding contributions to fluorescence and Raman emission in integrated photonic waveguides. Opt Express. 2025;33(22):45631–43. 10.1364/OE.575183 41414064

[44] Chen X, Andrews MP. Polarized and evanescent guided wave surface-enhanced Raman spectroscopy of ligand interactions on a plasmonic nanoparticle optical chemical bench. Biosens (Basel). 2024;14(9). 10.3390/bios14090409 PMC11430309 39329784

[45] Jensen MN, Gates JC, Flint AI, Helleso OG. Demonstrating low Raman background in UV-written SiO2 waveguides. Opt Express. 2023;31(19):31092–107. 10.1364/OE.498795 37710637

[46] Sharma A, Li Y, Prasad MK, Ho WL, Chu ST, Borzenets IV. Raman signal enhancement via a micro-ring resonator. Appl Phys Lett. 2025;126(5). 10.1063/5.0245521

[47] Zhao H, Teigell Beneitez N, Jena HS, Skirtach AG, Van Der Voort P, Baets R. Sensing of ceftazidime antibiotics in human plasma through waveguide-enhanced Raman spectroscopy assisted by a mesoporous layer. APL Photon. 2025;10(7). 10.1063/5.0278252

[48] Gao D, Liu J, Liu X, He K, Ma Z, Liu H, et al. Emerging frontiers in SERS-integrated optical waveguides: advancing portable and ultra-sensitive detection for trace liquid analysis. Light Sci Appl. 2025;14(1):389. 10.1038/s41377-025-01989-6 PMC12658145 41298381

[49] Makela M, Tu D, Lin Z, Cote G, Lin PT. Chip-scale aptamer sandwich assay using optical waveguide-assisted surface-enhanced Raman spectroscopy. Nanomaterials (Basel). 2024. 10.3390/nano14231927. PMC11643483 39683314

[50] Cameron JM, Mchardy RG, Sala A, Butler HJ, Palmer DS, Mitchell PJ, et al. A multiomic liquid biopsy for the earlier detection of colorectal cancer. Cancer Prev Res. 2026;19(3):145–52. 10.1158/1940-6207.capr-25-0286. PMC13012238 41431387

[51] Horta-Velázquez A, Arce F, Rodríguez-Sevilla E, Morales-Narváez E. Toward smart diagnostics via artificial intelligence-assisted surface-enhanced Raman spectroscopy. TrAC Trends Anal Chem. 2023. 10.1016/j.trac.2023.117378.

